# A Review of the Conceptualisation and Risk Factors Associated with Treatment-Resistant Depression 

**DOI:** 10.1155/2017/4176825

**Published:** 2017-08-03

**Authors:** Jenifer A. Murphy, Jerome Sarris, Gerard J. Byrne

**Affiliations:** ^1^ARCADIA Research Group, Professorial Unit, The Melbourne Clinic, Department of Psychiatry, University of Melbourne, Richmond, VIC, Australia; ^2^NICM, School of Health and Science, Western Sydney University, Campbelltown, NSW, Australia; ^3^Discipline of Psychiatry, School of Medicine, The University of Queensland, Brisbane, QLD, Australia

## Abstract

Major depression does not always remit. Difficult-to-treat depression is thought to contribute to the large disease burden posed by depression. Treatment-resistant depression (TRD) is the conventional term for nonresponse to treatment in individuals with major depression. Indicators of the phenomenon are the poor response rates to antidepressants in clinical practice and the overestimation of the efficacy of antidepressants in medical scientific literature. Current TRD staging models are based on anecdotal evidence without an empirical rationale to rank one treatment strategy above another. Many factors have been associated with TRD such as inflammatory system activation, abnormal neural activity, neurotransmitter dysfunction, melancholic clinical features, bipolarity, and a higher traumatic load. This narrative review provides an overview of this complex clinical problem and discusses the reconceptualization of depression using an illness staging model in line with other medical fields such as oncology.

## 1. Introduction

Refractory or treatment-resistant depression (TRD) refers to depression that is nonresponsive to treatment. The term “treatment-resistant depression” first appeared in medical scientific literature in the 1970s and has superseded “refractory depression” as the overarching label for nonresponse to treatment. The burden of depression is increasing [1] despite advancements in the safety and tolerability of treatments for depression over the past 50 years. The introduction of SSRIs in the 1980s as safe and viable treatments for depression created the illusion that depression was easily treatable and managed by antidepressant therapy. However, in more recent times, researchers and clinicians have shifted their view from depression as a treatable, acute illness to a chronic and recurrent illness that does not always respond to treatment [2].

Our current armamentarium of treatments for depression may not be as successful or efficacious as reported in randomised controlled trials (RCTs). There are long-standing concerns about publication biases which inflate the perceived efficacy of antidepressants in RCTs and inadvertently influence evidence-based care for individuals with depression [3, 4]. Clinical trials are also compromised by the placebo effect and the exclusion of patients who are treatment-resistant or who have a higher chance of nonresponse [5]. An analysis of unsuccessful and unpublished clinical trial data from the US FDA reports a symptom reduction rate of 42% for antidepressant trials, indicating that antidepressants may not be as effective as reported in medical scientific literature [6].

### 1.1. Prevalence of TRD

In an attempt to better understand the efficacy of antidepressants, the National Institute of Mental Health (NIMH) funded the Sequenced Treatment Alternatives to Relieve Depression (STAR^*∗*^D) study using a community representative sample of outpatients with MDD. The well-known STAR^*∗*^D study, which recruited over 4000 depressed outpatients in the USA, is the most comprehensive and representative view of the nonresponse of treatment for depression. Utilising a representative sample, the STAR^*∗*^D highlights the lower than expected efficacy of treatments for depression and the need for sequential treatments following the nonresponse to initial treatment in a majority of patients with depression [12]. A further implication of the STAR^*∗*^D study was the acknowledgement that patients with chronic or recurrent episodes of depression require a greater number of treatment strategies to potentiate response and have poorer long-term outcomes [12]. In depressed outpatients, the STAR^*∗*^D reports a cumulative remission rate of 50% after two different treatments are trialled [12, 13]. However, others estimate that between 60% and 70% of individuals with a major depressive illness do not achieve complete remission from their symptoms after receiving adequate treatment (standard antidepressant dose for an adequate duration, usually 6 weeks or more) [14]. Not surprisingly, lower levels of TRD are reported in primary care settings, whereas higher rates of TRD occur in inpatient psychiatric settings [15].

The lack of a standardised definition of TRD without a systematic way to identify the phenomenon in clinical practice and research has made prevalence estimates of TRD difficult [15]. Current prevalence estimates differ depending on the employed definition of TRD. Additionally, prevalence estimates are dependent on the treatment setting and study design used. In particular, how treatment outcomes and transitional points across the illness course (e.g. response, remission and relapse) are defined directly influence how TRD is conceptualised. This is because current definitions of TRD are reliant on predetermined symptomatology cut-offs and response criteria. Thus the need to conceptualise and empirically validate points across the depression illness course is paramount in order to adequately conceptualise and standardise the phenomenon of TRD.

### 1.2. Conceptualisation and Staging Models of TRD

Presently, the conceptualisation of TRD and its operationalisation in research and clinical practice are consensus driven rather than data driven [16]. This is because much about TRD is unknown and our ability to empirically test definitions is limited by heterogeneous research methodology and inconsistent findings [17]. This has delayed the translation of research findings into clinical practice and has impeded the development of new treatment strategies aimed at improving the outcomes of patients who are resistant to treatment.

Earlier systematic reviews investigating the definitional concepts surrounding TRD noted that depression is considered resistant when an individual fails to achieve a significant clinical improvement after receiving two antidepressant trials [16]. Findings from the STAR^*∗*^D which report a cumulative remission rate of 50% after two different treatments are trialled provide empirical support for the most commonly employed definition of TRD as the failure of two antidepressant trials [16]. The failure of two antidepressants is currently the most commonly used definition in medical scientific literature but has been criticised as oversimplifying the concept of TRD [18].

As a result, several staging models have been developed in order to stage individuals on a continuum of treatment resistance [7–10, 19, 20]. However, these models have not yet been appropriately validated and not one has been adopted for widespread use by researchers and clinicians. The five models have not been evaluated against one another in the same study. One study tested the validity of two TRD models [21]. The Thase and Rush Model (TRM) [7] and the Massachusetts General Hospital Staging method (MGHS) [9] were found to be highly correlated with one another but the MGHS demonstrated significantly greater ability to predict nonremission in individuals with MDD (*N* = 115) who were treated and assessed at academic specialty clinics over a 3-year period [21]. All available models appear to stage TRD arbitrarily without an empirical rationale for their particular staging method. This approach is explained by Trivedi et al. [22], who admit that models are based on algorithms of experience, expertise, and anecdotal impressions rather than empirical data because data simply do not exist and much about TRD is still unknown. Staging models are only useful for clinical practice when they are based on the latest evidence-based strategies. For example, many of the staging models assume that switching antidepressants is an effective strategy for treating resistant depression. However, recent evidence now suggests that switching is no more effective than persisting with the ineffective antidepressant for a longer duration [23]. A few of the most notable staging models are shown in Table 1.

## 2. Factors Associated with TRD

Previous studies investigating the factors associated with treatment resistance have not been consistently replicated and are limited by research and sample heterogeneity. Other factors such as misdiagnosis, individual clinician differences, comorbidity, inadequate treatment, and patient heterogeneity are all considered to contribute to treatment resistance under the banner of “pseudoresistance.” Pseudoresistance refers to treatment resistance as a result of diagnostic and/or treatment factors which when remedied may actually result in treatment responsive depression and better patient outcomes. However, these factors do not explain the phenomenon of TRD in its entirety.

The underlying aetiology of depression has been widely studied with many different theories proposed. Furthermore, the DSM-IV and DSM-5 are atheoretical as to the cause of depression [24]. Applying the many theories to a unified aetiological model of depression has been difficult, as only selected theories apply to certain types of depression and to particular points across the illness course [25]. Even less clear is how the many theories of depression apply to treatment response.  Table 2 highlights some of the factors associated with TRD.

### 2.1. Biological Correlates

The biological base of depression and any neurobiological differences that might exist between treatment responsive and treatment-resistant depression remain unclear. There are reported differences in brain structure and function, as well as, molecular differences in TRD patients in comparison to healthy controls and nonresistant depressed patients.

#### 2.1.1. Neuroendocrine and Immune Systems

It is widely acknowledged that depression is associated with immune suppression and immune activation [26]. A bidirectional relationship between inflammation and depression is thought to exist [27]. Particular attention has been given to cytokines, cell signalling proteins that mediate and regulate immune response, and depression [27]. Proinflammatory cytokines promote the inflammatory response while anti-inflammatory cytokines work to reduce inflammation and initiate healing [27]. Efforts to identify neuroendocrine and immune dysfunction in depression have focused on alterations in hypothalamic-pituitary adrenal (HPA) regulation and other neuroendocrine changes such as elevated cortisol levels as well as altered immune function [25, 28].

Hyperactivity of the HPA axis is thought to be activated by the proliferation of inflammatory cytokines [27]. An increase in proinflammatory cytokines has been associated with HPA axis disturbance and is thought to lead to the release of the stress-hormone, cortisol [27]. Cortisol has a long-standing association with depression with cortisol reported to be elevated in depressed patients [27]. Furthermore, overactivity of the HPA axis in depression is supported by findings which suggest that chronic imipramine treatment (tricyclic antidepressant) downregulates the plasma levels of important hormones involved in the HPA axis thus highlighting the role of the HPA axis in depression and immune dysfunction [27, 29].

In studies comparing treatment-resistant samples to controls, HPA axis disturbance [30], proliferative activity of T cells [31], and overall activation of the inflammatory system [30, 31] have been associated with TRD. However, elevated basal cortisol levels have not been reported in TRD inpatients (*N* = 36) in comparison to healthy controls (*N* = 31) [29]. Despite the unexpected lack of reported increases in basal cortisol in TRD patients, inpatients with TRD have shown altered immunoneuroendocrine regulation due to glucocorticoid-induced suppression of lymphocyte proliferation (e.g., T cells) in comparison to the healthy controls [29]. This finding suggest that immune function and steroid regulation in TRD patients may be associated with lymphocyte steroid resistance rather than elevated levels of cortisol as previously reported in depression [29].

Treatments for depression can help elucidate the role specific biological correlates might play in depression. Coenzyme Q10 (CoQ10), which is synthesised from the amino acid tyrosine, is hypothesised to have anti-inflammatory effects and has been studied as a potential treatment for TRD [32]. Low CoQ10 levels in depression may indicate a greater inflammatory response [32]. In line with previous findings, which associate TRD with greater activation of the inflammatory system, lower plasma CoQ10 has been linked to TRD and also to individuals with depression and comorbid chronic fatigue syndrome [32]. Thus, CoQ10 supplementation may conceivably provide benefit as an adjunct to treatment for resistant depression [32]. However, to date, no randomised controlled trials have been conducted to confirm the efficacy of CoQ10 as a treatment for depression.

#### 2.1.2. Neural Systems and Circuits

HPA axis disturbance along with prolonged exposure to glucocorticoids and/or stress-induced reductions in neurotrophic factors may result in the reduced hippocampal volume commonly linked to depression [33]. Furthermore, a reduced or small hippocampal volume has also been identified as a risk factor for depression and treatment resistance [33]. The volume of other brain structures, such as the entorhinal cortex, which has reciprocal connectivity with the hippocampus, has also been reported as reduced in TRD patients in comparison to healthy controls (*N* = 15 versus 17, resp.) [34]. However, this effect was only found in females and not males [34]. The brain reward system which includes structures in the nucleus accumbens septi (NAcc) and the superolateral branch of the medial forebrain bundle (slMFB) have been identified as potential targets for deep brain stimulation to treat TRD [35]. The reward system has long been associated with depression and addiction with white matter abnormalities within the medial forebrain bundle associated with TRD characteristics such as anhedonia, melancholic features, and symptom severity [35].

Neural circuitry within specific neural systems mediates stress responsiveness, mood, and emotional regulation [36]. The use of a perfusion magnetic resonance imaging (MRI) technique known as arterial spin labelling (ASL) has found hyperfusion regions in the bilateral subgenual anterior cingulate cortex (sACC), left dorsomedial prefrontal cortex, and left subcortical areas (putamen, pallidum, and amygdala) in TRD patients compared to healthy controls [37]. Hyperactivation of the sACC provides evidence for dysfunctional cortical circuits in depression [37]. The subgenual cingulate region has been previously implicated in modulating negative mood states and also in antidepressant treatment response [38].

Other circuits such as the limbic-cortical-striatal-pallidal-thalamic circuit [39] and the prefrontal-amygdalar-pallidostriatal-mediothalamic circuit [40] have been implicated in TRD in comparison to healthy controls. The limbic-cortical-striatal-pallidal-thalamic circuit closely resembles the default-mode network, a system of brain regions (medial prefrontal cortex, posterior cingulate/retrosplenial cortex, and left and right inferior parietal lobules) that show decreased activation during goal-oriented or attention-demanding tasks [41]. The default-mode network is associated with episodic memory, self-reflection, and emotional regulation [42, 43].

A recent study used voxel-based morphometry of structural and fMRI data to compare the concentrations of gray matter in the default-mode network regions between TRD (*N* = 18), treatment responsive depression (*N* = 17), and healthy controls (*N* = 17) [43]. Resting-state functional connectivity analysis was also conducted to investigate the gray matter abnormalities between the groups [43]. Both the TRD and treatment responsive depression groups showed significant gray matter abnormalities in the right middle temporal cortex and bilateral caudate. Furthermore, patterns of resting state functional connectivity in these areas were different between all three groups [43]. Alterations in functional connectivity in different brain regions between TRD and treatment responsive patients were found. In particular, the regions of aberrant connectivity were mainly located in the default-mode network [43]. This finding provides evidence for the default-mode network's likely involvement in the pathophysiology of depression [43].

A second study used structural MRI, voxel-based morphometry, and multivariate pattern analysis in an attempt to classify TRD patients (*N* = 18), patients with first-episode MDD (*N* = 17), and healthy controls (*N* = 17) [44]. Differing patterns of gray matter and white matter volumes in the areas of the brain regions associated with the default-mode network significantly discriminated between TRD patients, patients with first-episode MDD, and healthy controls [44]. However, because only subtle differences in functional connectivity were found between TRD and treatment responsive patients in both studies, it is unclear what role the default-mode network plays in treatment response. Notwithstanding, it is clear that sensitive neuroimaging methods may have greater utility in identifying subtle alterations in the default-mode network associated with treatment response [44].

#### 2.1.3. Neurotransmitter Dysfunction

Theories of neurotransmitter dysfunction in depression are well established. The predominant theory hypothesises that depression is related to decreased availability of monoamine neurotransmitters. In more recent times the monoamine theory of depression has shifted from a theory of depleted monoamines (particularly noradrenaline and serotonin) to the integration of a theory of neurotransmitter dysfunction resulting from an interaction between stressful life events and the serotonin transporter gene [45, 46]. A gene-by-environment interaction is theorised where a functional polymorphism of the serotonin transporter gene moderates the influence of stressful life events in people with depression [45]. However, a recent meta-analysis comprising 14 studies found no evidence of the serotonin transporter gene interacting with stressful life events to increase the risk of depression [47].

Current antidepressants act on multiple monoamine neurotransmitters and have targeted effects on neurotransmitter function [46]. However, the response to these conventional antidepressants is delayed and often unsatisfactory [46, 48]. The poor response to antidepressants has led to suggestions that the monoamine theory of depression does not fully explain neurotransmitter dysfunction in depression. Other neurotransmitters and systems may contribute to the dysfunction and perceived treatment resistance [48]. In particular, the glutamatergic system has garnered significant attention [48].

Gamma-aminobutyric acid (GABA) is the major inhibitory neurotransmitter in the central nervous system and balances neuronal excitability produced by glutamate [48]. The efficacy of antiglutamatergic agents (such as lamotrigine and ketamine) for the treatment of depression provides support for excessive glutamate induced excitation in depression [48]. In relation to treatment resistance, lower levels of GABA in the occipital cortex have been found in medication-free TRD outpatients (*N* = 15) in comparison to healthy controls (*N* = 24) and medication-free treatment responsive depression outpatients (*N* = 18) [49]. Furthermore, deficits in GABA_A_ and GABA_B_ receptor-mediated inhibitory neurotransmission distinguished TRD (*N* = 25) from healthy controls (*N* = 25), medicated previously depressed patients (*N* = 19), and unmedicated currently depressed patients (*N* = 16) [50]. Therefore, marked GABAergic deficits may be characteristic of TRD, suggesting the possible usefulness of therapeutic strategies aimed at potentiating cortical GABA in patients with TRD (e.g., lamotrigine augmentation, electroconvulsive therapy, and transcranial magnetic stimulation) [50]. There is also increasing evidence of the potential usefulness of ketamine, a glutamate N-methyl-D-aspartate (NMDA) receptor antagonist which works in part through glutamate release onto the AMPA receptor, as a treatment for TRD [51].

### 2.2. Genetic Correlates

Advances in genetic epidemiology have spurred research investigating the role genetics play in the pathophysiology of depression. Researchers have studied the genetics of the serotonin transporter (5-HTT) located on the presynaptic neuron as a way to investigate the serotonergic system [52]. As many antidepressants target serotonin reuptake mechanisms, the serotonin transporter is a popular site to study the role genetics play in treatment response. Response to treatment in depression is considered to be associated with signalling through 5-HT1A receptor and with neurogenesis in the hippocampus [53]. Differences in response to treatment have been linked to polymorphisms in the 5-HTT promoter region (5HTTLPR) [54]. The presence of particular environmental factors, together with a specific expression of genes, may leave individuals vulnerable to depression and poorer treatment response.

The Group for the Study of Resistant Depression (GSRD) conducted a large candidate gene study to assess phenotypes associated with antidepressant treatment response [55]. A single nucleotide polymorphism (SNP), rs20755865, was associated with antidepressant treatment response in the GSRD sample [55]. Other treatment response phenotypes in the GSRD sample were found in the brain-derived neurotrophic factor (BDNF) (rs10501087 and rs6265),* 5HTR2A* (rs7997012) and* CREB1* (rs7569963) genes [55]. The BDNF gene is an important candidate for research as BDNF has been implicated in brain plasticity and antidepressant treatment response [56]. Serum BDNF levels are thought to increase in response to antidepressant treatment [56]. BDNF is thought to function through its high affinity receptor, neurotrophic tyrosine kinase receptor 2 (NTRK2) [56]. Interactions between BDNF (rs6265) and NTRK2 (rs1387923, rs2769605, and rs1565445) have been found, providing further support that BDNF levels in the brain may play an important role in antidepressant treatment response [56].

### 2.3. Psychological and Psychosocial Correlates

Treatment resistance in depression has commonly been linked to an earlier age of onset [57, 58], more frequent [57, 59] and recurrent [58] episodes of depression, longer duration of illness [59], greater severity of depression [58], and an older current age [59]. Patients with TRD are also more likely to be hospitalised for treatment [58] and to have a greater risk of suicide [58–60]. Nonremission or partial remission after a previous depressive episode [57] and nonresponse to the first antidepressant ever trialled [58] have also been identified as potential risk factors for TRD.

There has been evidence to suggest that TRD is associated with the “melancholia” subtype of depression as a high prevalence of the subtype has been found in TRD outpatients [17, 58]. The melancholia subtype has historically been distinguishable from other types of depression by disturbances in affect, which are disproportionate or without cause, psychomotor retardation, cognitive impairment, and vegetative dysfunction [61]. Depressed patients classified with the melancholic subtype are less likely to respond to placebos and psychotherapies [62] and are more responsive to tricyclic antidepressants [63] and ECT [64]. Furthermore, two recent studies [32, 65] found that TRD was associated with a symptom profile similar to that of melancholia subtype, with symptoms such as anhedonia [65], suicidal thoughts [65], concentration difficulties [65], autonomic disturbances [32], and sleep disturbances [32, 65] characterising TRD in comparison to controls or treatment responsive depression.

Higher rates of both psychiatric and general medical comorbid disorders have been reported in association with TRD [58, 66]. In terms of psychiatric comorbidity, TRD has been associated with a higher prevalence of comorbid anxiety disorders [58], panic disorder [58], social phobia [58, 67], and personality disorders [58]. As cited in Fava [9], moderate consumption of alcohol has been associated with poorer response to treatment. Alcohol and/or substance use may complicate the presentation of TRD and should be evaluated and treated alongside the resistant depression.

TRD has also been linked to a possible bipolar diathesis [57]. However, the presence of high levels of bipolarity symptoms in TRD samples could be due to the presence or history of antidepressant-induced hypomania. A retrospective chart audit of 146 TRD patients found evidence of treatment induced hypomania or hypomanic-like episodes in a small number of TRD audited cases (*N* = 16) [68]. The link between bipolarity and TRD raises the possibility of pseudoresistance due to misdiagnosis. However, these studies report bipolarity features, not proof per se of comorbid bipolar disorders. Subclinical bipolarity features or treatment induced hypomania rather than bipolar comorbidity may be associated with TRD.

Patients with TRD are reported to experience a higher number of stressful life events, including immigration, death of a family member, interpersonal relationship problems, job loss, financial stress, severe health conditions, and life-threatening situations [66]. In a recent study, adverse childhood experiences including trauma and bullying were reported as common in TRD (defined as the failure of one antidepressant) with 62% of TRD inpatients (*N* = 137) reporting childhood adversity [69]. An early study which used the Thase and Rush [7] model of TRD to define treatment resistance reported high levels of trauma and emotional abuse in TRD patients compared to non-TRD patients [70]. The authors conclude that early trauma may result in an increased vulnerability to life stressors in patients with TRD [70].

#### 2.3.1. Personality Traits and Treatment Response in Depression

The relationship between personality and depression extends beyond the risk, onset, and maintenance of the disorder and has been implicated in treatment response [71–74]. In the broadest sense, personality dysfunction as measured by the Standardised Assessment of Personality-Abbreviated Scale (SAPAS) has predicted poorer short-term (6 weeks) response to antidepressant treatment in a large sample of depressed outpatients (*N* = 8229) [71]. Reviewing the five-factor model and treatment response, a large systematic review (*N* = 50 studies) identified high neuroticism as a predictor of worse treatment outcomes particularly over a long-term follow-up period [74].

While there have been various studies investigating personality and treatment response in depression, there have been very few studies assessing personality in depressed samples employing a standardised definition of TRD. A brief report by Kaplan and Klinetob [70] found higher scores on the Minnesota Multiphasic Personality Inventory (MMP1-2) subscales (all except hypomania) in outpatients with TRD compared to individuals with non-TRD [70]. A more recent study assessed the personality profile of patients with TRD (*N* = 35) compared to patients with remitted depression (*N* = 27) and healthy controls (*N* = 66) using the five-factor model [75]. The definition of TRD employed was the nonresponse to at least two antidepressants [75]. The TRD sample had significantly higher neuroticism and lower extraversion, openness, and conscientiousness scores on the NEO-PI compared to healthy controls and patients with remitted depression (i.e., not TRD) [75]. The authors propose that low openness may be a feature unique to TRD and may be related to lower levels of resilience [75]. This is in line with the conclusions presented by Kaplan and Klinetob [70] who propose that TRD outpatients may be more vulnerable to perceiving life stressors as traumatic and have “fewer psychological defences” and lower levels of resilience to manage these stressors.

Low openness in the TRD sample was positively associated with cooperativeness and reward dependence on the Temperament and Character Inventory (TCI) [75]. The constructs of cooperativeness and reward dependence are self-reported styles of social behaviour. Individuals with low cooperatives are thought to be socially intolerant, disinterested in other people, alienated, hostile, unhelpful, and revengeful [76]. Furthermore, higher levels of social inhibition, as measured by the Social Inhibition (SI) Scale, have been associated with TRD [67]. It has been suggested that socially inhibited individuals may not be able to create and maintain the social networks needed to moderate life stress and depression [67].

Identification of a unique personality profile or maladaptive personality functioning in TRD could help to assist in identifying TRD patients in clinical practice, provide insight into the onset and maintenance of TRD, and assist clinicians in tailoring psychological treatments for this severely affected group of patients.

## 3. Discussion

Similarities and differences between individuals who respond to treatment and those who do not can provide insight into the aetiology of TRD. However, due to the major heterogeneity in research methods the correlates of TRD have not been consistently replicated and have been difficult to distinguish from major depression more generally. Furthermore, due to cross-sectional nature of many studies it is not clear whether these correlates are risk factors or consequences of TRD. This is an inherent flaw of most cross-sectional research with longitudinal studies providing much needed clarity in the field of depression and treatment response [77].

In order to study any phenomenon or illness state it must be labelled and defined in a way that is operational. Since the 1970s, the nonresponse to treatment for individuals with depression has been acknowledged and labelled first as treatment refractory depression and later as TRD. Despite naming the phenomenon and acknowledging its existence over 40 years ago, the field of psychiatry has not settled on how to define it and, more importantly, how to operationalize it. This is not for want of trying. There have been many attempts to standardise the concept of TRD using either a dichotomous definition of the failure of two antidepressants or by staging TRD on a continuum of resistance. However, no single model has been adopted for widespread use by researchers and clinicians. Additionally, nonpharmaceutical treatments for depression (e.g., psychotherapy, ECT, TMS, and VNS) are not included in many models of TRD. Thus, the models fail to fully encompass the complete phenomenon of nonresponse to treatment [77].

There has been a rise in the number of RCTs conducted in patients with TRD in recent years. Despite this growing interest in developing new treatment strategies for TRD patients, the findings are difficult to interpret and replicate due to major variations in the operationalisation of TRD from study to study. Nonclinical trial data on TRD are less common and there are a limited number of naturalistic cohort or case-control studies, which investigate the phenomenon. Additionally, there has been no clear consensus on why or how some patients become treatment-resistant. Even though risk factors for TRD and theories of resistance have been put forward in medical scientific literature we are no closer to understanding the aetiology of TRD and no closer to prospectively identifying which patients are likely to be poor responders to treatment. Clinical prediction models have not been successful at identifying TRD in clinical populations, suggesting that other, unmeasured, variables (e.g., endophenotypes) are likely to be involved in treatment resistance.

Why is the phenomenon of nonresponse to treatment so difficult to conceptualise and operationalize? One partial explanation could be that TRD is not diagnosable as a distinct disorder in the DSM-5 or ICD-10 and therefore open to continual interpretation and conceptualisation. Alternatively, the failure to conceptualise and operationalise TRD in a clinically meaningful way could be linked to how we conceptualise depression more generally. The DSM-5 proposes that depression occurs in discrete episodes which, when treated effectively, results in a return to premorbid functioning and wellness. However, this is not the case for a large proportion of patients. Depression is likely to recur and in almost one-third of patients it follows a chronic illness trajectory [78]. In recent times, there has been a shift in psychiatry, acknowledging that depression is not as treatable and episodic as once thought. Furthermore, our current treatments for depression appear to be no more effective than they were 50 years ago despite ongoing research efforts [77].

It could be argued that we have outgrown our current diagnostic classification for depression because it no longer adequately reflects what we know about the disorder and how it is treated. Main revisions to the conceptualisation of depression in the past 35 years have been the abolishment of neurotic versus endogenous depression in the ICD-10 and the removal of the bereavement exclusion from the DSM-5. The removal of the bereavement exclusion may have a considerable impact on the conceptualisation of depression by failing to delineate normal sadness from clinical depression or sadness without cause. However, this is yet to be seen. Even prior to the removal of the bereavement exclusion, there were growing concerns that heterogeneous presentations of depression were being fitted into a homogenous diagnostic classification system largely ignoring aetiology and symptom clusters representing depression subtypes, for example, melancholia and atypical depression. As a consequence, TRD has developed its own heterogeneity with resistance to treatment occurring for multiple reasons, at different points during the illness course and to specific treatments only. An additional caveat of depression research is the constant struggle between calling for more standard treatment selection to systematically assess treatment efficacy and the recognition that different symptom clusters (or depression subtypes) require different treatments. The conceptualisation of the disorder, as the endorsement of one or two core symptoms (depressed mood or anhedonia) alongside four or more other depression symptoms is arbitrary and creates major heterogeneity in clinical presentations [77].

Without the reconceptualization of depression, both treatment approaches cannot occur simultaneously. Staging depression in a similar way to medical diseases such as cancer or infectious diseases may provide the opportunity to systematically guide treatment selection based on clinical presentation and the progression of the disorder (see Table 1). Several prominent clinicians and researchers have put forward illness staging models of depression [11, 79]. McGorry et al.'s [11] model defines each illness stage, as well as potential interventions, relevant patient populations, and indicative endophenotypic markers for psychotic and severe mood disorders. It is the most comprehensive model to date. However, McGorry et al. [11] do not incorporate neurobiological findings which characterise the progression of psychiatric disorders from the prodromal stage to Stages 4 and 5 [80]. This has become increasingly important as evidence suggests that recurrent and chronic depression states result in inflammation, oxidative stress, and loss of neurotrophic factors leading to potentially irreversible neuronal circuit damage and functional and structural brain atrophy [80]. This inevitability will be the focus of future research and refinement of staging models going forward. It is not yet clear whether these more comprehensive models will be adopted for use in clinical practice and research.

## Figures and Tables

**Table 1:** The evolution of TRD staging models.

Thase and Rush Model [7]	European Staging Model [8]	Massachusetts General Hospital Staging Model (MGHS) [9]	Maudsley Staging Model [10]	McGorry – Clinical staging framework [11]		
*Stage I*: failure of 1 AD of 1 major class of AD *Stage II*: failure of 2 AD of at least 2 different classes of ADs *Stage III*: Stage II plus failure of a TCA *Stage IV*: Stage III plus failure of a MAOI *Stage V*: Stage IV plus a course of bilateral ECT	*Stage A, nonresponder*: nonresponse to 1 AD or ECT with a trial duration of 6 to 8 weeks (score = 1) *Stage B, TRD*: TRD1, resistance to 2 or more ADs of different classes for at least 12- to 16-week duration (score = 2) TRD2, resistance to 2 or more ADs of different classes for at least 18- to 24-week duration (score = 3) TRD3, resistance to 2 or more ADs of different classes for at least 24- to 32-week duration (score = 4) TRD4, resistance to 2 or more ADs of different classes for at least 30- to 40-week duration (score = 5) TRD5, resistance to 2 or more ADs of different classes for at least 36- to 52-week duration (score = 6) *Stage C, CRD*: resistance to several AD trials (at least 5) including augmentation strategies (score = 7) Total score = 1 to 7	*Stage I*: nonresponse to each adequate (at least 6 weeks at an adequate dose) trial of an AD (1 point per trial) *Stage II*: optimisation of dose, optimisation of duration, and augmentation or combination of ach trial (increases overall TRD score by 0.5 for each optimisation/augmentation) *Stage III*: ECT used (3 points) Total score = summation of each treatment trial	*Duration*: acute (≤12 mnths) = 1 point; subacute (13 to 24 mnths) = 2 points; chronic (>24 mnths) = 3 points *Symptom severity*: subsyndromal = 1 point; mild = 2; moderate = 3; severe without psychosis = 4; severe with psychosis = 5 *Treatment failures*: Level 1: 1-2 ADs = 1 point Level 2: 3-4 ADs = 2 points Level 3: 5-6 ADs = 3 points Level 4: 7–10 ADs = 4 points Level 5: >10 ADs = 5 points Augmentation: not used = 0 points; used = 1 point ECT: not used = 0 points; used = 1 point Total score = 3 to 15	*Stage 0*: increased risk; no symptoms currently	*Intervention* Psychoeducation; brief cognitive skills training	*Markers* For example, smooth pursuit eye movements, mismatch negativity,
				*Stage 1a/b*: mild or nonspecific symptoms or mild functional change or ultrahigh-risk.	Psychoeducation; CBT; substance use reduction; antidepressants	For example, folate status, HPA axis dysregulation
				*Stage 2*: first episode. Moderate to severe severity and functional decline	Psychoeducation; CBT; substance use reduction; antidepressants	Continue with markers of illness state and progression
				*Stage 3a*: incomplete remission from first episode. Could fast track to Step 4	As for 2 with emphasis on medical and psychosocial strategies to achieve remission	Continue with markers of illness state and progression
				*Stage 3b*: recurrence or relapse or residual symptoms	As for 3a with emphasis on relapse prevention and early warning signs of relapse	Continue with markers of illness state and progression
				*Stage 3c*: multiple relapses	As for 3b with emphasis on long-term stabilization	Continue with markers of illness state and progression
				*Stage 4*: Severe, persistent or unremitting illness	As for 3c, but with emphasis on augmentation strategies	Continue with markers of illness state and progression

(i) No definition of dose/duration of trials (ii) Ranks antidepressant classes hierarchically (iii) Ordinal staging	(i) TRD stages and the distinction between TRD and CRD are arbitrarily chosen based on trial duration and number of treatment failures (ii) Ordinal staging	(i) Each trial is scored individually resulting in a continuous scale with no maximum total (ii) No antidepressant hierarchy (iii) Incorporates augmentation and ECT	(i) Multidimensional model includes illness duration, severity, augmentation and ECT (ii) No antidepressant hierarchy (iii) Continuous staging	(i) Entire illness progression staged (ii) Biomarkers incorporated (although does not include genetic or neurobiological markers) (iii) Targeted interventions for each stage		

AD, antidepressants; CBT, cognitive behavioural therapy; CRD, chronic resistant depression; ECT, electroconvulsive therapy; HPA, hypothalamic-pituitary-adrenal; MAOI, monoamine oxidase inhibitor; MGHS, Massachusetts General Hospital Staging Model; TCA, tricyclic antidepressants; TRD, treatment resistant depression.

**Table 2:** Summary of the biological, psychological, genetic, and clinical correlates of TRD.

*Biological*
Activation of the inflammatory system
HPA axis disturbance
Dysfunctional neuroanatomic circuits (particularly the default mode network)
Abnormal neural activity
Neurotransmitter dysfunction

*Clinical and psychosocial*
Melancholic features
Frequent and recurrent episodes
Previous nonremission or partial remission
Long illness duration/chronicity
Prevalence of psychiatric co-morbidity
Bipolarity features
High number of stressful life events/trauma

*Genetic*
Involvement of polymorphisms in the 5-HTT promoter region (5HTTLPR)
Interactions between BDNF and NTRK2 polymorphisms

*Personality*
Personality dysfunction
High neuroticism
Low extraversion, openness and conscientiousness
High levels of social inhibition

BDNF, brain derived neurotrophic factor; NTRK2, neurotrophic tyrosine kinase receptor 2.
